# Impact of Music Therapy on Neurodevelopment of Preterm Infants and Functional Improvement in Children with Neurological Deficits

**DOI:** 10.3390/pediatric17020041

**Published:** 2025-03-28

**Authors:** Filomeni Armakola, Eleni Potamiti, Anna Tsiakiri, Georgios Felekis, Georgia Tsakni, Athanasios Tsivgoulis, Christos Moschovos, Sotirios Giannopoulos, Vasiliki Georgousopoulou, Markos Sgantzos, Pinelopi Vlotinou

**Affiliations:** 1 Early Rehabilitation Department of Children’s Hospital P. & A. Kyriakou Hospital, 11527 Athens, Greece; farmakola@uniwa.gr (F.A.); epotamiti@gmail.com (E.P.); 2 Neurology Department, Democritus University of Thrace, 68100 Alexandroupolis, Greece; atsiakir@med.duth.gr; 3 General Medicine English Section, University of Medicine and Pharmacy “Grigore T. Popa”, 700115 Iasi, Romania; sikelef1@gmail.com; 4 Occupational Therapy Department, University of West Attica, 12243 Athens, Greece; ytsakni@uniwa.gr; 5 Attika Rehabilitation Center, 19018 Magoula, Greece; nasost02@yahoo.gr; 6 Second Department of Neurology, Atiikon Hospital of Medicine, National and Kapodistrian University of Athens, 15772 Athens, Greece; sgiannop@uoi.gr (S.G.); moship@windowslive.com (C.M.); 7 Department of Nursing, University General Hospital of Alexandroupolis, 68100 Alexandroupoli, Greece; vgeorgousopoulou@yahoo.gr; 8 Department of Anatomy, Faculty of Medicine, University of Thessaly, 41110 Larissa, Greece; sgantzos@med.uth.gr

**Keywords:** music therapy (MT), biomarkers, neonates, children, neurodevelopment, functionality

## Abstract

Background/Objectives: The aim of this systematic review is to assess the effect of music therapy initiated during neonatal intensive care unit (NICU) hospitalization on the early neurodevelopment of infants and to evaluate its impact on functional improvements in children with neurological deficits. Numerous studies underscore the benefits of neurological music therapy (NMT) for treating various neurological conditions. Methods: This systematic review (SR) specifically includes randomized controlled trials (RCTs) and draws from a comprehensive search of articles in the Scopus and MEDLINE databases. Eligible studies examined the effects of NMT on infants and children with central nervous system static lesions. Eighteen studies met all inclusion criteria, and the overall quality of the evidence was high. Results: However, while NMT appears to be well-tolerated by most neonates and children and holds promise for enhancing functional and physiological development, its impact on specific biomarkers in neurological conditions remains underexplored. Further research is essential to clarify NMT’s potential role in rehabilitation and to optimize therapeutic approaches for neurological support.

## 1. Introduction

The term “biomarker” is derived from “biological marker” and refers to a broad category of objective measurements that assess a patient’s health with precision and consistency. According to the World Health Organization (WHO), a biomarker is “any substance, structure, or process measured in the body or its products that influence or predict the incidence of outcome or disease”. The WHO further defines biomarkers in the context of environmental risk assessment as “almost any measurement reflecting an interaction between a biological system and a potential hazard, which may be chemical, physical, or biological” [1].

One field that increasingly explores the role of biomarkers is music therapy (MT), a research-supported practice in which professionals use music to address physical, emotional, cognitive, and social needs. Through activities such as listening to music, singing, playing instruments, and composing, MT enhances communication, improves motor skills, and fosters emotional expression. It is widely applied in hospitals, schools, rehabilitation centers, and mental health institutions to support individuals of all ages. Music therapy (MT) has emerged as a significant therapeutic modality over the past decade, drawing interest from researchers and clinicians alike for its broad applications across various health domains. It is particularly noted for its efficacy in addressing both physiological and neurological conditions, making it a promising intervention for pediatric populations due to its safe and non-invasive nature [2]. MT uses the inherent qualities of music to elicit a variety of psychological, emotional, and physical reactions, providing a comprehensive, well-tolerated, and non-invasive therapeutic approach [3,4].

Previous studies have shown that MT can affect infants’ physiological biomarkers, such as heart rate (HR), respiratory rate (RR), and oxygen saturation—oftentimes severely affected by physiological stress—as well as feeding volume, infant behavior, and reduction of parental anxiety [5,6,7]. In medical terms, a newborn or neonate (from the Latin neonatus, meaning newborn) is an infant in the first 28 days after birth (the term applies to premature, full term, and postmature infants) [8]. These effects are particularly relevant in pediatric populations, where stress and anxiety are prevalent and in which conventional pharmacological interventions may pose risks or have limited effectiveness [9]. Research is ongoing in this particular direction, and it is important to note that, to the best of our knowledge, no published studies have looked into how MT affects the physiological parameters of children and adolescents, who cannot—due to age or cognitive state—respond to self-reported psychometrically validated questionnaires for stress and anxiety. This particular population could be evaluated through stress biomarkers, a method already used in adults [10,11]. MT’s ability to induce relaxation and alleviate stress is well-documented, with mechanisms believed to involve the parasympathetic nervous system’s activation and reduction in cortisol levels [12,13,14]. Τhe application of music and/or musical components (such as harmony, rhythm, and sound) to achieve objectives like stress reduction or life quality enhancement can be applied in different ways. For preterm infants, we noted that music was mostly accessed as recorded lullabies, the works of Mozart, or mother’s live lullabies, while for children, instrumental music and piano lessons were the main uses of music. The duration of MT is more adaptable. It can last from 10 min to 1 h, two to four times per week, from 4 week to 18 months, and even each day for infants in the neonatal care unit, for 2 weeks or until their discharge, to promote better neurodevelopment. While several systematic reviews have explored the efficacy of MT in specific contexts, such as for pain management, stress reduction, and cognitive enhancement, few have synthesized these outcomes to provide a holistic understanding of MT’s therapeutic potential [15,16]. The rhythmic and melodic elements of music are thought to synchronize with physiological rhythms, promoting homeostasis and reducing the physiological burden of stress [14].

Another aspect in which MT has been shown to have potential for becoming a staple supplementary therapeutic module is for promoting functional improvements in patients with neurological conditions. Music is believed to promote neuroplasticity, through feedback and feedforward mechanisms of information transmission, engaging both sensory and motor areas of the brain [17,18,19,20,21]. Neuroimaging studies have revealed that MT can lead to structural changes in the brain regions involved in memory, attention, and executive function, thereby enhancing cognitive performance [22,23]. In older patients with stroke or neurological degenerative diseases, such as Parkinson’s, MT has proven to be effective in promoting individuals’ physical and cognitive abilities, in addition to their general quality of life [24]. The effect of MT on children and youth with neurological diseases that affect motor skills has not yet been thoroughly investigated, yet it is possible to use music assist in improving motor skills, particularly fine and gross motor coordination, which are crucial for the development of functional independence in children with neurodevelopmental disorders [25,26]. These improvements are often attributed to the rhythmic components of music, which can enhance sensorimotor integration and facilitate the reorganization of motor pathways [27]. The evidence of increased neuroplasticity, as indicated by neuroimaging and electrophysiological studies, further supports the potential of MT to influence the brain’s structure and function, which may underlie the observed improvements [28,29,30,31]. The aim of our study is to observe the impact of music therapy on the physiological markers and functionality of the pediatric population.

## 2. Materials and Methods

### 2.1. Search Approach

A thorough search of the literature was conducted to find pertinent studies on the effects of music therapy (MT) on physiological (blood pressure, oxygen saturation, heart rate) markers of pediatric patients with static lesions of the central nervous system of prenatal or perinatal origins, with manifestation such as cerebral palsy and/or cognitive disorders, as well as its effects on neurological outcomes in children and adolescents. The following databases were searched: MEDLINE, Pub Med Central, and Scopus. The search covered the period from 5 November 2023, to 27 December 2023, with Prospero registration number CRD42024583202. Search terms included a mix of terms from MeSH and free-text terms such as “music therapy”, “biomarkers”, “children”, “neurological disorders”, “clinical trials”, and “randomized controlled trials (RCTs)”.

### 2.2. Study Selection

**Figure 1:**
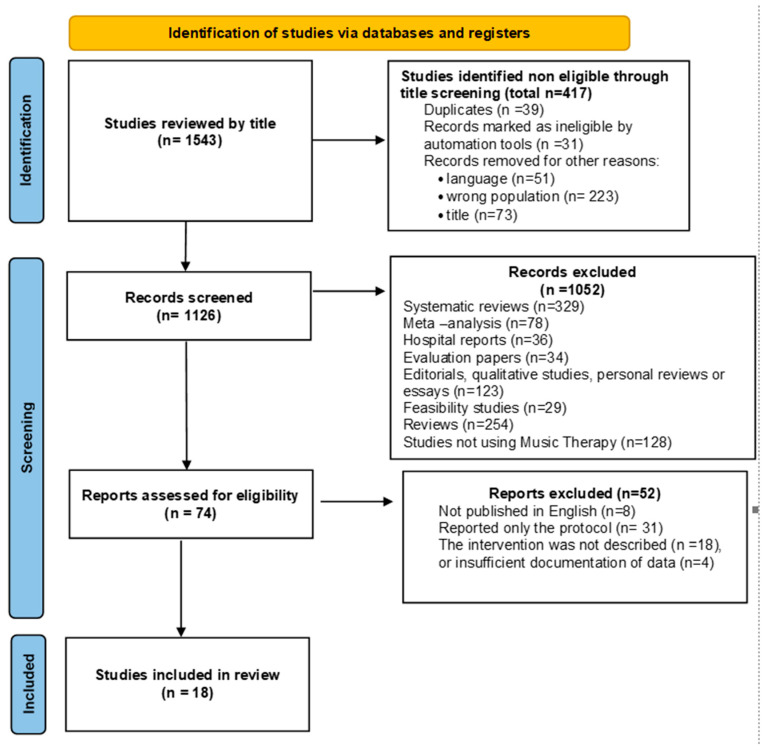
PRISMA flow diagram for systematic review including searches of the data bases.

All search results were imported into EndNote 21 software to manage citations and remove duplicates. Two independent reviewers (A.F. and A.T.) screened the titles and abstracts for relevance to the research question. Disagreements among reviewers were settled by conversation, and a third reviewer (V.P.) was consulted if consensus could not be reached, in cases where the intervention was not thoroughly described. Studies were included if they comprised RCTs with at least single-blinding, involved pediatric populations (0–18 years), and investigated the impact of MT on physiological or neurological outcomes. The PRISMA (Preferred Reporting Items for Systematic Reviews and Meta-Analyses) [32] flow diagram in Figure 1 outlines the study selection process.

### 2.3. Inclusion and Exclusion Criteria

Inclusion criteria were as follows: clinical studies or RCTs published in peer-reviewed journals within the last 15 years, written in English, and involving pediatric participants aged 0–18 years. Additionally, studies without a clearly defined therapeutic intervention were excluded. The rationale for excluding other study types was to ensure the inclusion of high-quality evidence and reduce potential bias.

### 2.4. Data Management and Extraction

A standardized data extraction form was used to extract the data, which included variables such as population characteristics, sample size, type of intervention, frequency and duration of MT sessions, outcomes measured, and potential biases. Extracted data were double-checked for accuracy by a second reviewer. The extracted data were then summarized, focusing on key outcomes such as HR, RR, oxygen saturation, motor and cognitive skills, and neuroplasticity—the neural system’s ability to create and rearrange synaptic connections, particularly in response to experience, learning, or after injury in order to gain more, less, or modified lost functions.

### 2.5. Evaluation of Quality

The PEDro (Physiotherapy Evidence Database) scale was used to evaluate the methodological quality of the included research. This scale was chosen because it provides a quick assessment of the reliability and feasibility results of the tested interventions for clinical practice, which cover the scope of this review. The tool evaluated various aspects of study design, including randomization, blinding, and completeness of outcome data, as shown in Table 1.

## 3. Results

### 3.1. Study Selection

The systematic search found 1543 records. After removing duplicates, 1126 unique articles remained. Title and abstract screening led to the exclusion of 1052 studies that did not meet the inclusion criteria. Full-text reviews were conducted for the remaining 74 articles, of which 57 were excluded based on criteria such as study design, population, and outcome relevance. In the end, 18 studies were included in the evaluation, after meeting the inclusion criteria. The PRISMA flow diagram is included in (Figure 1).

### 3.2. Study Characteristics

The final set of 18 studies, published between 2007 and 2022, collectively involved 1354 participants, whose ages ranged from neonates to 18 years. The studies were geographically diverse, conducted across North America (n = 4), Europe (n = 7), and Asia (n = 7). The primary outcomes examined included physiological parameters such as HR, RR, and oxygen saturation, as well as neurological outcomes, including cognitive function, motor skills, and neuroplasticity. Sample sizes varied, with individual studies enrolling between 16 and 108 individuals. The interventions spanned 4 weeks to 18 months, with most studies implementing weekly music therapy (MT) sessions of 30 to 60 min. The characteristics of each study are summarized in Table 2.

### 3.3. Risk of Bias

Quality evaluation using the PEDro scale indicated that 15 studies were of high quality (PEDro score ≥ 6), while the remaining 3 studies were of moderate quality (PEDro score 4–5). The principal source of bias was related to blinding, particularly in cases where blinding of participants and therapists was not feasible due to the nature of the MT intervention. Bias attributed to non-blinding affects the validity and reliability of results, as it can lead to misinterpretations of the available data. Additionally, three studies exhibited a significant chance of attrition bias, mostly as a result of insufficient outcome data. The PEDro scoring is displayed in Table 1.

### 3.4. Preterm Neonates

Six publications that address the impact of music on preterm babies are included in our review. Only one clinical trial offered MT to the entire cohort [33], yet its intervention design is based on a therapeutic rubric that interchanges two different music modules and the control (no music), in six separate sequences, suggesting that all infants received MT sessions. All of them measured biomarkers such as O_2_ saturation, heart rate, respiratory rate, and among their secondary outcomes, they measured days of hospitalization, the average daily weight increase, the gestational age at discharge, the number of days until complete feeds were achieved, and the number of days receiving IV feeding.

The length of intervention was set between 20 min [33,36] and 30 min [36,37]. The only study setting a firm time frame for the beginning of the intervention was Menke et al. [36], who proposed the 21st day of life. The frequency of sessions between the clinical trials varied greatly, from (at least) one day/week [33], to everyday interventions for 14 consecutive days [36]. Two studies found that MT improves O_2_ saturation and decreases both heart and respiratory rate [35,37]. On the other hand, the outcomes of those specific parameters showed no statistically significant differences in a third study [38], posing a difficulty for reaching a safe conclusion concerning the efficacy of the interventions regarding physiological parameters in the NICU. An interesting conclusion comes from the study by Walworth et al. [34], who noted in their results that infants in their intervention group spent fewer days in the hospital compared to the controls (12.9 days on average) and needed less time to integrate feeding behavior. On a similar note, another study found that MT intervention groups required a shorter duration of all forms of therapy compared to controls, a positive result that was combined with the reduction in parental stress [36].

In summary, regarding premature infants, the hypotheses that music promotes biomarkers and physiological development is partially confirmed.

### 3.5. Children and Adolescents

We found no clinical trial that studied the effect of MT on children between >4 months and 4–5 years old with motor impairment due to neurological deficit. Also, as mentioned previously, the key outcomes for the efficacy of MT in infants and school-age children and adolescents are usually completely different. The usual choices for infants are physiological parameters, whereas for older children, measurements focus on cognitive and motor function. Interestingly, our research found one recent paper, bridging the gap between the two age categories, in which hospitalized children received MT as a supplement to their physical therapy (PT) sessions twice a week, resulting in significantly reduced HR and RR and elevated O_2_ saturation levels in the intervention group compared to the results for the controls [39].

In our search, the main cluster of research found focused on motor functionality in youths. The main tools used to measure the outcomes were a grip dynamometer, the Gross Motor Function Measure (GMFM), the Gross Motor Function Classification System (GMFCS), the Manual Ability Classification System (MACS), and the Box and Block assessment tools, as well as the PEDI Daily Mobility scale. The time frame and frequency of MT interventions varied greatly from one study to the other, as did the type of intervention chosen in each case, posing an added difficulty for a safe comparison, e.g., in one study, researchers opted for an intervention of 30–45 min/twice a week, stretching out over a period of 18 months [40], whereas in another, the intervention was based on 10–30 min sessions, four times a week, for 4 weeks [41].

The outcomes were encouraging in the vast majority of studies, reporting improvement in hand grip strength and gross and fine motor skills, along with self-reported improvement in everyday functionality [21,41,42,43,44]. Moreover, it is crucial to note that the findings of one study, which recruited young people with cerebral palsy (CP) for the longest intervention duration (18 months), could not support the hypotheses of the positive outcomes of MT in enhancing functionality, as no significant changes in grip strength or finger agility were observed in their selected cohort [40].

Two papers studied the cognitive outcomes of music therapy in children with intellectual deficiency using different MT methods, targeting linguistic organization, attention, and general cognitive skills. In both cases, the results showed that MT can improve auditory processing skills [45,46], and in one of them, parents reported the improved social communication of their children [46].

Perhaps the most interesting findings in our systematic review come from three studies that aimed to investigate whether MT can have an effect on brain neuroplasticity [47,48,49]. Using different methods—functional magnetic resonance imaging (fMRI), event-related desynchronization (ERD), and magnetic resonance imaging (MRI) [47]—they found positive correlations between MT and brain activity. Alves-Pinto et al. [48] reported positive connectivity of the left motor cortex area to the right cerebellum after 18 months of piano lessons in youths with neuromotor impairments, results that are in concordance with the results of a study that included youths and adults with CP who took piano lessons twice a week for 4 weeks. In this study, alpha ERD oscillations were correlated with improved hand agility and strength [45]. A third study that supports the hypotheses of MT enhancing brain plasticity is one that studied children with motor and cognitive impairments. Researchers designed a rather intensive, short-term program of 10 min sessions, three times/day, 3 days/week for 4 to 8 weeks, depending on the child. They reported improved abilities in regards to focusing and communication skills, as well as modifications in brain plasticity, in the intervention group [47]. The study by Bringas et al. evaluated the family of mismatch responses (MMR), which is the differential change of event-related brain potentials (ERP) to “deviant stimuli” integrated in a series of “standard stimuli”, as a promising electrophysiological substitute for assessing brain plasticity. Both normal processes and brain diseases can be detected by the highly sensitive MMR biomarkers. During MT, the right prefrontal cortex and the bilateral medial cingulate cortex were activated, improving attention-related functions, resulting in positive MMR changes. These findings suggest that MT can be a useful supplementary therapy to enhance brain plasticity, which, when combined with other therapeutic methods that target directing neuroplasticity to produce tangible functional improvements, can enhance the therapeutic outcomes.

## 4. Discussion

Changes in measures of brain activity and brain structural modifications are also considered biomarkers. However, different indicators were measured across studies, depending on the population examined. Specifically, studies on neonates predominantly focused on physiological biomarkers such as oxygen saturation, heart rate, and respiratory rate, while studies on older children emphasized neuroplasticity and functional brain activity changes. Given these distinctions, our review presents findings separately for these two groups to ensure clarity and appropriate interpretation of the results.

Regarding premature infants, our findings suggest that MT interventions such as recorded lullabies, Mozart selections, or mother’s live lullabies could improve O_2_ saturation, heart and respiratory rate, and feeding volume, as well as decrease the total length of hospital stays and reduce parental stress. The literature review reveals that music therapy appears to exert its strongest physiologic impact in decreasing neonates’ heart rate, with the majority of systematic reviews and meta-analyses suggesting its positive effect [6,50,51,52,53,54,55]. Like all auditory stimuli, music is communicated by air vibrations, which the cochlea then converts into electrical impulses. Nerve impulses caused by sound waves are transmitted from the cochlea to the brain for interpretation.

Numerous neural response attributes encode a wide range of sound characteristics, which are then transferred to the auditory brainstem, which consists of the superior olivary complex and the inferior colliculus. These brainstem areas lead to the auditory thalamus, which in turn leads to the main auditory cortex (A1) via the ventral geniculate body. Listening to music stimulates A1, as well as motor and pre-motor areas, including the cerebellum, primary motor areas, supplementary motor areas, and basal ganglia, because music is a multimodal stimulus. When listening to music, the frontotemporal–cerebellar circuit’s A1 connection provides perceptual processing. On the other hand, processing the emotional content of music is linked to A1 co-activation with the motor, pre-motor, insula, and cerebellum. The integration of working memory, linked to the temporal dynamics of the sound, is made possible by another significant functional loop between the A1 and inferior frontal areas, especially in the right hemisphere [3].

However, if the data are viewed while keeping in mind that neonate HR displays a wide expected variability [54], the clinical significance of the findings might be uncertain [6].

In addition, due to the small sample size of most studies, there are reasonable concerns regarding the generalizability and statistical power of the data provided. Furthermore, interventions included in the present study varied greatly in regards to most variables (delivery methods, beginning and total number of sessions, duration of each session, and frequency), posing challenges in the interpretation of the extracted data. The only studied parameters that are consistent throughout the whole volume of the available literature reveal that music therapy is safe and feasible in the NICU setting.

In the pediatric and adolescent population, our study found that MT can improve hand grip strength and gross and fine motor skills, marking the positive correlation with increased brain activity, which could in turn be correlated with improved hand agility and strength results, suggesting that MT might help enhance brain plasticity in the developing brain. A recent systematic review found that auditory stimulation can improve gait and postural management in children with CP by enhancing their spatiotemporal and kinematic control [56]. In other words, rhythm, which is an essential music component, can co-activate the brain regions involved in motor planning and execution [4,57,58], indicating that auditory stimuli alone can improve both gross and fine motor functions. The recent systematic reviews by Yang et al. [19] and Yanagiwara et al. [54] indeed proved that MT can enhance motor skills in children with CP. Additionally, in children and youth with cognitive deficits due to neurological conditions, this systematic review found that MT can improve auditory processing and communication skills. These results are in concordance with other systematic reviews recently published, which highlight the effects of MT—oftentimes significant—on social, emotional, and cognitive skills [59,60,61,62,63].

### Limitations and Future Directions

While the findings are promising, it is important to recognize several restrictions. There was a great deal of variation among the included studies in terms of intervention protocols, duration, and outcome measures, which may affect the generalizability of the results. Although sample size in the studies included with MT interventions on neonates in the NICU could be described as adequate, the variability that certain biomarkers (i.e., heart rate) present in said age group requires caution in order to avoid the possibility of a logical fallacy due to hasty generalizations. Another crucial factor for determining the efficacy of MT in infants is the methodological inconsistency between studies, with variations in MT durations, frequencies, methods, starting points, and duration of the studies. In addition, not all interventions were delivered by accredited MT specialists.

Regarding MT interventions on children and youth, studying the efficacy of such programs using the functional results achieved, the samples could be deemed as small, and there was heterogeneity between recruited groups, not only in their underlying pathology, but also regarding their baseline functionality levels, rendering a comparison between risky. Variability in the methods used, timelines for data collection, frequency, and duration between the program, further impede the efforts to achieve a more critical analysis.

Furthermore, most of the research was carried out in wealthy nations, potentially restricting the applicability of these findings to more diverse populations. Future research should focus on standardizing MT protocols to facilitate comparisons across studies and exploring the long-term effects of MT. Clearly defined protocols implemented on larger and more specific populations, as well as studies examining the intensity of the programs (duration of sessions and interventions) and type of MT, or its combination with PT, OT, and SLT, are needed to clarify the method’s effectiveness. Additionally, there is a need for studies conducted in nations with lower and middle economic status to determine the global applicability of MT.

## 5. Conclusions

In conclusion, this systematic review provides a valuable overview, showing that simple biomarkers (blood pressure, oxygen saturation) and tests with no cost (walking, hands ability) can be integrated (by all members of a rehabilitation team) during rehabilitation programs by incorporating music therapy to evaluate the progress of infants or children. Moreover, this study demonstrates that music therapy can be offered as a valuable tool to enhance both physiological and neurological outcomes in children and adolescents, given its non-invasive, low- cost nature and its ability to promote neuroplasticity. These findings highlight MT as a viable and effective therapeutic intervention that can be integrated into pediatric healthcare. Continued research is necessary to refine MT interventions, assess their long-term efficacy, and expand their applicability across different cultural and clinical settings and specific pediatric populations.

## Figures and Tables

**Table 1:** PEDro scale score for clinical trials included in the review. Green indicates that the study meets the specific PEDro criterion, while red indicates that the study does not meet the criterion.

	SCALE CRITERIA	Elimination Criteria	Random Distribution	Hidden Distribution	Homogeneity	Blind Subjects (Patients)	Blind Therapists	Blind Assessor(s)	Measured More than >85% of Patients	“Intention to Treat”	Comparison Between Groups	Measures of Variability	T O T A L
Study													
Marrades-Caballero et al., 2018 [21]		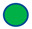	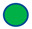	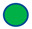	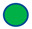	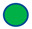	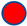	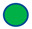	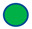	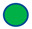	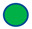	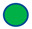	10
Amini et al., 2013 [33]		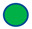	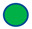	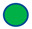	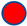	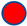	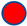	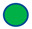	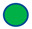	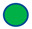	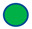	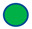	8
Walworth et al., 2012 [34]		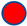	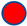	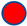	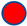	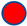	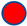	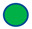	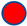	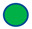	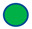	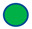	4
Namjoo et al., 2022 [35]		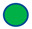	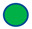	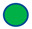	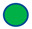	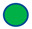	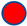	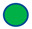	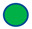	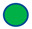	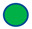	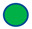	10
Menke et al., 2021 [36]		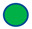	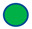	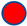	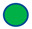	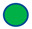	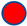	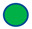	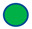	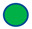	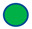	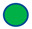	9
Jabraeili et al., 2016 [37]		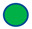	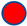	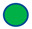	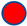	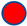	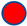	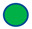	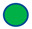	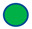	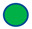	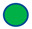	7
Alipour et al., 2013 [38]		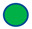	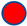	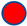	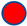	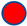	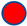	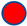	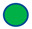	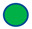	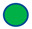	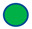	5
Kobus et al., 2022 [39]		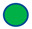	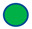	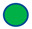	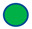	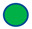	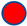	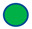	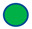	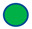	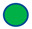	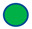	10
Lampe et al., 2015 [40]		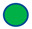	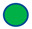	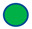	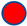	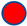	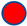	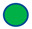	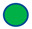	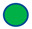	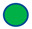	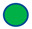	8
Ben-Pazi et al., 2018 [41]		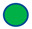	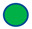	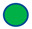	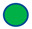	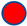	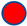	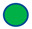	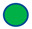	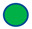	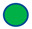	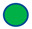	9
Dogruoz karatekin et al., 2021 [42]		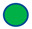	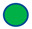	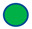	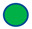	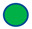	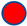	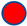	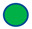	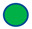	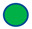	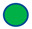	9
Wang et al., 2013 [43]		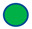	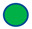	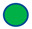	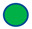	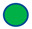	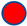	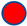	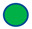	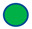	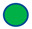	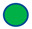	9
Teixeira-Machado et al., 2016 [44]		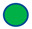	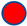	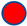	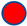	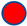	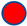	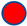	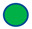	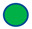	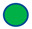	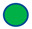	5
Senkal and Muhtar 2021 [45]		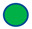	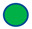	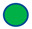	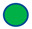	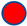	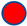	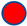	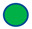	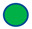	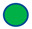	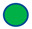	8
Sharda et al., 2018 [46]		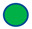	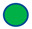	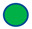	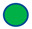	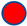	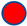	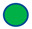	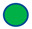	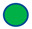	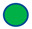	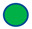	9
Bringas et al., 2015 [47]		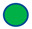	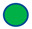	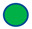	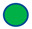	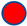	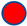	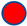	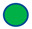	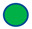	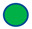	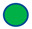	8
Alves pinto et al., 2015 [48]		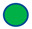	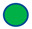	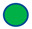	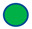	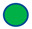	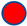	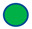	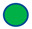	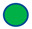	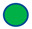	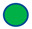	10
Alves pinto et al., 2017 [49]		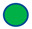	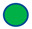	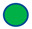	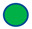	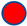	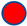	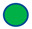	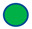	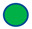	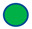	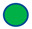	9

**Table 2:** PICO table of music- based interventions.

Study	Population	Intervention	Control Group	Outcome Measures	Measurements	Results
Alipour et al., 2013 [38]	n = 90 premature infants in incubators n = 30, music group n = 30, silence group, n = 30, control group	A total of 30 min after feeding and other routine care, earphones and sensors were placed, and a lullaby was played for 20 min. In the silence group, headphones did not play music.	Usual care	O_2_ saturation, respiratory rate, heart rate, and behavioral state of the infants	Data recorded at the 5th, 10th, 15th, and 20th minutes of intervention and at the 5th and 10th minutes post-intervention.	Lullaby music failed to induce significant alterations in the mean values of O_2_ saturation, respiratory and heart rates, and behavioral state of infants.
Alves-Pinto et al., 2015 [48]	n = 16 youth n = 10, MT n = 6, control group	Individualized piano lessons for 18 months (mean age: 12.8).	No training during the same period of time (mean age: 16.4)	Functional imaging of the brain.	Data obtained before the start of the training and 18 months later, after the “piano” group had finished the training.	Increase in positive connectivity from the left primary motor cortical area to the right cerebellum for the MT group.
Alves-Pinto et al., 2017 [49]	n = 22 n = 9 teenagers, CP, 11–17 y.o. n = 6 control group, typical teenagers n = 7 adults, CP, 34–52 y.o.	Twice a week with professional piano teacher for 4 weeks. Total 8 h training at school or care center. Typical population: teens receive training at home. For ten of the participants (controls and patients), training was supported by a technical system specially developed to assist patients with sensorimotor deficits. Hand motor tests (piano tests). Vibration tests on fingers. Alpha “ERD’’ to assess neuronal correlation with motor learning.		Hand motor tests (piano tests): Vibration tests on fingers Alpha “ERD’’ to assess neuronal correlation with motor learning.	First experimental session was followed by a second experimental session 4 weeks later, with no piano training in between. After the second experimental session, piano lessons were introduced for 4 weeks, leading to the third experimental session.	A significant effect of training on the ability to perceive the localization of vibrations over fingers was noted. No effects of training on the performance of simple finger tapping sequences at the piano or on motor-associated brain responses were registered.
Amini et al., 2013 [33]	n = 25 neonates Six groups with A (lullaby), B (Mozart), C (control group), interchanging in six sequences: ABC, ACB, BAC, BCA, CAB, and CBA.	RCT cross-over design intervention sequence in six groups an hour after feeding. A: 10 min of initial observation, B: music (lullaby or Mozart, based on a predetermined sequence) starting at 45–50 dB for 20 min, C: video recording for 10 min after the music stopped.	Usual care	O_2_ saturation, heart rate, respiratory rate	Video data collected for 40 min in three phases: baseline, intervention phase, and post intervention phase.	O_2_ saturation, when compared to baseline, did not change during the intervention phase. HR in lullaby group in all intervention phases was significantly reduced, but in “Mozart group”, reduction was only observed during the intervention phase. Reduction in RR was observed both in intervention and post intervention phases in all groups. Results for lullaby during both phases and for Mozart during post intervention phase were statistically significant.
Ben-Pazi et al., 2018 [41]	n = 18, paired children (age: 7y 5m, SD 4y 1m; 13 boys; GMFCS: median 4; MACS: median 4)	Auditory stimulation embedded in music. At least 10 min up to 30 min four times a week for 4 weeks.	Music listening	Goal Attainment Scale, Care and Comfort Hypertonicity Questionnaire, Gross Motor-Function Measure, and Quality of Upper Extremity Skills Test (QUEST)	First session and 5 months following intervention.	Vast improvement in function in individual children, especially in walking, standing, Care and Comfort (CCHQ), Goal Attainment Scale (GAS) and Quality of Upper Extremity Skills Test (QUEST). Compliance was similar between the two groups.
Bringas et al., 2015 [47]	n = 34 children with significant problems regarding motor, cognitive, and specifically, in communication, abilities. n = 17 MT, “Auditory Attention plus Communication Protocol” n = 17, control group	Before the usual occupational and speech therapy (10 min sessions immediately before the standard speech and occupational therapies), three times a day, 3 days per week, over 4 or 8 weeks, depending on the duration of therapy. This resulted in a total of 36 sessions of MT (360 min) after 4 weeks and 72 sessions of MT (720 min) after 8 weeks.	Usual occupational and speech therapy	Special purpose questionnaire that incorporated several different well-established procedures and behavioral outcomes. Brain plasticity changes in MT Reflected by ERP mismatch responses (MMR).	At T0 and the end.	Improved attention and communication, as well as changes in brain plasticity in children with severe neurological impairments, only in the experimental group. Changes in brain plasticity also occurred in the experimental group. LORETA EEG source analysis identified prefrontal and midcingulate regions as differentially activated by MT in the experimental group.
Dogruoz and Icagasioglu, 2021 [42]	n = 18 n = 9, adolescent cerebral palsy n = 9, healthy adolescent volunteers (control group)	Therapeutic instrumental music performance method was applied 2 days a week for 3 months in 40 min sessions.		MACS, Box Block Test, Nine-Hole Peg Test, Jamar hand dynamometer strength, and key pressing force of fingers were evaluated with Cubase MIDI program.	Before and after 3 months of training.	With the therapeutic instrumental music performance method, functional gains were achieved in the grip strength, strength of the fingers, and gross and fine motor skills of adolescent cerebral palsy patients.
Jabraeili et al., 2016 [37]	n = 66 premature infants, 29–34 weeks g.a., <2800 gr n = 25, Brahm’s lullaby n = 21, mum’s lullaby n = 20, control group	Timing of patterned sound exposure was 3 consecutive days, with the intervention implemented between 10:00 A.M. and 7:00 P.M., with the exception of the time between two shifts.	Usual care	O_2_ saturation	O_2_ saturation was recorded continuously for 45 min (10 min before, 15 min during, and 20 min after the sessions).	There were significant differences in neonate O_2_ saturation between the Brahm’s lullaby and Mum’s lullaby groups as compared with the control groups at the 15 min point after intervention.
Kobus et al., 2022 [39]	n = 17 hospitalized children, age 0–18	SG music therapy during the physical therapy session twice a week. CG physical therapy session twice a week, without music therapy. The mean duration of each therapy session was 44 min (range 21 and 71 min)		Heart rate, respiratory rate, and oxygen saturation.	15 min before to 15 min after session	Music therapy supports the children in physical therapy interventions during their hospitalization. We observed significantly lower heart and respiratory rates and higher oxygen saturation during physical therapy intervention with live music therapy, in general.
Lampe et al., 2015 [40]	n = 18 children and youths n = 10, CP n = 8, global retardation and movement coordination disorder (GRMCD)	All children continued their regular therapy program (physiotherapy twice a week and swimming therapy once a week) during the study. A total of 30–45 min of piano training with a professional piano teacher twice a week for 18 months.		Manual Ability Classification System for description of manual skills in CP, Box and Block test, and dynamometer test.	Before and after the training.	The average results of the Box and Block test before and after piano training showed an improvement. No significant changes in grip strength were registered. For both CP and GRMCD groups, the average time interval between consecutive strokes remained practically unchanged throughout the training period.
Menke et al., 2021 [36]	50 parent–infant pairs, infants born at <30 weeks of g.a. n = 24, MT group n = 26, control group	The treatment group received music therapy twice a week from the 21st day of life until discharge from hospital, 20–30 min/session.	Usual care	Physiological development at discharge time and stress, anxiety, and postpartum depressive symptoms, as well as an increase in parental skills as primary caregivers over the time period from baseline.	At the 21st day of infant’s life (baseline) and at the day of discharge.	At time of discharge, preterm infants in the treatment group showed descriptively shorter durations of all forms of therapy compared to preterm infants in the control group. No significant difference when comparing all physiological variables between the two groups. Therapy durations were shorter in the study group (fewer days: on caffeine therapy, on nasogastric/orogastric tube feed, in hospital), but the development factor did not differ between the groups. From pre-to-post-intervention, parents showed a significant reduction in stress factors, with no statistical difference between two groups.
Marrades-Caballero et al., 2018 [21]	n = 18 children with severe bilateral cerebral palsy between 4 and 16 years old	Therapeutic instrumental music performance (TIMP) for 40 min per session once a week for 16 weeks, in addition to physical therapy, for a total of 13 sessions.		Chailey Levels of Ability	T0 beginning and the end.	Significant improvements in the overall and specific “arm and hand position”, as well as “activities” from the Chailey Levels of Ability, and in the locomotor stages, were observed (*p* < 0.05) in the group which received music therapy. All these improvements persisted after 4 months. The control group showed no improvements after a 4-month follow-up.
Namjoo et al., 2022 [35]	90 preterm infants: n = 30, live lullaby n = 30, recorded lullaby n = 30, no intervention	Music (recorded lullabies and mother’s live lullabies) was played for 14 days, 20 min a day.	Usual care	Heart rate, O_2_ saturation, sleep	A total of 10 min before intervention, during intervention, and 20 min after intervention.	Τhere was an improvement in O_2_ saturation and a decrease in HR in the two intervention groups, but no statistical difference was noted between groups. The mean duration of the infants’ overnight sleep was not statistically significant between the groups before the intervention, but there was a statistically significant difference in the intervention groups after the intervention; the infants’ overnight sleep was longer in the recorded-lullaby group than in the other two groups.
Senkal and Muhtar, 2021 [45]	N = 58 n = 29, study group with intellectual deficiency n = 29, control group	Two 45 min sessions per week for 6 weeks.		TLI- linguistic organization (LO) attention, sensory, motor, social and behavioral skills, decoding/language mechanics, auditory processing scores.	Beginning and end of the trial.	The mean musical assessment scores improved after Orff Music Therapy. The TLI scores were reduced after Orff Music Therapy, which means there was an improvement in auditory processing skills.
Sharda et al., 2018 [46]	n = 51 n = 26, study group: blinded, parallel-group RCT n = 25, control group no MT	Individual 45 min weekly sessions conducted over 8–12 weeks by the same accredited therapist (M.T.)	Usual care	ADOS, Social Responsiveness Scale, the children’s communication cognitive ability, language ability by sentence repetition, and receptive vocabulary.	Baseline assessment in two sessions with MRI.	MT can improve parent-reported social communication, FqoL, and intrinsic brain connectivity in school-age children, thus supporting the use of music as a therapeutic tool for individuals with ASD.
Teixeira-Machado et al., 2017 [44]	n = 26 CP, 15–29 y.o.	Dance group: four sets of eight repetitions. ROM, motor coordination. Body image, interaction with environment, skill, and agility.	24 sessions (1 h, twice a week)	Functional Independence Measure (FIM) and World Health Organization Disability Assessment Schedule (WHODAS) by International Classification of Functioning, Disability, and Health (ICF).	Measured before and after each intervention.	Dance could have an influence on basic common points in the body and motion, including emotional and social aspects, supporting the concept of complex multimodal psychomotor adjustments. Dance promoted enhancement of functionality and social activities regarding psychosocial adjustments in young cerebral palsy subjects.
Walworth et al., 2012 [34]	108 preterm infants: n = 25, live singing n = 29, live singing and guitar n = 54, control group	Infants in the experimental group received developmental multimodal stimulation (DMS) sessions lasting 20 min at least once per week for the duration of their stay, along with tactile stimulation.	Usual care and placement in mothers’ arms.	Length of stay, average daily weight gain, gestational age at discharge, number of days to full feeds, number of days receiving IV nutrition.	At the start of the program and at discharge from the NICU.	Length of stay: 12.9 days less on average for the experimental group. No significant differences between groups in average daily weight gain and in gestational age at discharge. Infants in the intervention groups integrated feeding behaviors more quickly.
Wang et al., 2013 [43]	n = 36 n = 18, study group n = 18, control group, no MT		A 6-week, home-based, loaded sit-to-stand exercise, but only the PSE group exercised with pre-recorded PSE music; three times per week under the supervision of their caregivers.	GMFCS Outcome Measures. PEDI Daily Mobility and Self-Care Functions Measures.	Baseline (T0), after 6 weeks of training (T1), at 6 weeks (T2), and at 12 weeks following the end of the training (T3). All assessments were performed by four trained physical therapists who were blinded to the treatment allocation.	Children who exercised with PSE music showed statistically significant improvements in gross motor capacity compared to that of the controls, and such effects could last at least 3 months; however, the PSE music did not achieve statistically significant improvements in participants’ daily functioning, strength, and walking speed.

## Data Availability

No new data were created or analyzed in this study.

## Abbreviations

The following abbreviations are used in this manuscript:

NICU	neonatal intensive care unit
NMT	neurological music therapy
SR	systematic review
RCTs	randomized controlled trials
MT	music therapy
HR	heart rate
RR	respiratory rate
PT	physical therapy
GMFM	Gross Motor Function Measure
GMFCS	Gross Motor Function Classification System
MACS	Manual Ability Classification
CP	cerebral palsy
FMRI	functional magnetic resonance imaging
ERD	event- related desynchronization
MRI	magnetic resonance imaging
